# Improving outpatient satisfaction by extending expected waiting time

**DOI:** 10.1186/s12913-019-4408-3

**Published:** 2019-08-13

**Authors:** Wei-Min Ma, Hui Zhang, Neng-Li Wang

**Affiliations:** 1 School of Economics and Management, Tongji University, Shanghai, 200092 China; 2 The Center for Pediatric Liver Diseases, Children’s Hospital of Fudan University, Shanghai, 201102 China

**Keywords:** Expected waiting time, Unfavorable information, Satisfaction score, Actual waiting time, Prospect theory

## Abstract

**Background:**

Long waiting times result in low satisfaction. Although several methods are used to shorten the actual waiting time (AWT) in large hospitals of China, the outpatients still have a long actual waiting time. This study aimed to explore whether satisfaction could be improved by extending the expected waiting time (EWT) instead of shortening the AWT.

**Methods:**

In October 2016, 257 students in grade one voluntarily participated in this study. They came from 6 classes, which were randomly divided into two groups: 3 classes comprised the control group (*n* = 125) and 3 classes comprised the experimental group (*n* = 132). Unfavorable information (UI) was given to the experimental group alone. Six distinct questionnaires were designed to explore the effects of UI on EWT and the effects of an extended EWT on satisfaction. Satisfaction scores ranged from 0 to 100: 0–25, very dissatisfied; 26–50, dissatisfied; 51–75, satisfied; 76–100, very satisfied. Each participant finished one of the 6 questionnaires online. Of the 257 questionnaires, 233 were valid.

**Results:**

Before UI was given, the initial EWT (T_0_) was similar between the control and experimental groups (*Z* = -1.924, *P* = 0.054). Under the effects of UI, individuals in the experimental group extended their EWT (T_1_) from 121.0 to 180.0 min (*Z* = -6.367, *P* < 0.001). Females prolonged their EWT longer than males did (*Z* = -2.239, *P* = 0.025). Then, this study defined T_0_ = 1.5 h and T_1_ = 2.5 h, and compared the satisfaction scores between the control and experimental groups: a significant difference was found when AWT =2.0 h (*t* = − 3.568, *P* = 0.001), but not when AWT =3.0 h (*t* = − 0.718, *P* = 0.475) or when AWT =1.0 h (*t* = − 1.088, *P* = 0.280). When AWT =3.0 h, fewer individuals felt “very dissatisfied” in the experimental group (21.2%) than in the control group (44.7%) (*χ*^*2*^ = 4.368, *P* = 0.037).

**Conclusions:**

EWT was found to be extended greatly by UI. An extended EWT could improve satisfaction scores.

**Electronic supplementary material:**

The online version of this article (10.1186/s12913-019-4408-3) contains supplementary material, which is available to authorized users.

## Background

Patients in China prefer to seek help in large hospitals, where better medical equipment and specialists are available [1]. However, patients have to face long waits in outpatient departments of large hospitals. Long waiting times result in dissatisfaction [2]. Patient satisfaction is based on evaluation of their experiences in the hospital [3]. It is an important factor in evaluations of health care quality [4]. To improve patient satisfaction, in addition to hospitals’ responsiveness in improving nonmedical aspects of care [5], doctors are advised to spend more time with their patients [6]. However, longer patient-doctor contact times will further increase waiting times if the numbers of doctors and patients do not change. Several methods have focused on improving patient satisfaction by reducing the actual waiting time (AWT).

Appointment systems [7–10], Lean Six Sigma [11–13], and Lean methods [14–16] are usually used to reduce the AWT. With appointment systems, patients can make an appointment and then visit the doctor at the scheduled time. Appointments help to reduce the AWT and the number of patients waiting in outpatient departments [7]. However, they may cause losses for the hospital if the patients do not arrive for their appointments [17]. Lean Six Sigma helps to identify possible process failures [11]. By redesigning the process, AWT can be shortened, which improves patient satisfaction. Lean principles can also be used for process improvements [13]. When the Lean principles are applied, activities that do not provide services will be identified and eliminated gradually, and new ways to solve the problem will be used [18]. In addition, hospitals are also advised to shorten the AWT by motivating doctors properly [19] and strengthening cooperation among medical teams [20]. However, due to the shortage of medical resources and the increasing numbers of patients, patients still have to face long waits in the outpatient departments of large hospitals in China.

According to the prospect theory, reference point determines an individual’s value judgment and risk behavior [21]. For the same results, different reference points determine whether subjects will feel satisfied. The reference point forms before the final result occurs and can be adjusted by many factors, including culture and unfavorable information (UI) [22, 23]. UI can be defined as information that tends to produce an unfavorable outcome [24]. UI affects reference point adaptation and subsequent decision making [23]. An individual’s value judgment will change according to the adaptation of reference point. Expectation can be severed as a reference point [25]. It is an important factor in determining satisfaction [26]. Expected waiting time (EWT) is defined as the amount of time that patients expect to spend in the hospital. EWT is formed before the patient sees a doctor and changes throughout the hospital visit. EWT is associated with patients’ perceived value, which is an important antecedent of overall patient satisfaction [27]. UI may also have great effects on EWT. Because adjusting the AWT can only partially improve satisfaction, this study focuses on whether satisfaction can be improved by adjusting the EWT.

In outpatient departments of large hospitals in China, the number of patients is characterized by periodic fluctuations. The AWT is directly related to the number of patients: the more patients visiting the outpatient departments, the longer the waiting time. Therefore, the information that there are many patients in the outpatient departments today can serve as UI and can be provided to patients in advance. The purposes of this study are to investigate the effects of UI on EWT and to further explore the influences of EWT on satisfaction. Our research takes a first step forward in improving satisfaction by extending the EWT.

## Methods

### Experiment design

Study subjects were randomly divided into two groups: the control group and the experiment group. They were asked to finish our online questionnaire survey. At the beginning of the questionnaire, a scenario was presented to mimic a hospital visit. To analyze the effects of UI on EWT, UI was given to subjects in the experimental group but not the control group.

For the control group, the individuals were asked to state the length of time they expected to wait, which was defined as their initial EWT (T_0_). AWT has been found to have an important effect on patient satisfaction [11–16]. This study further confirmed the effects of AWT (T_a_) on patient satisfaction with a fixed EWT. Three different hypothetical conditions were presented: T_0_ = 1.5 h and T_a_ = 1.0 h in condition 1; T_0_ = 1.5 h and T_a_ = 2.0 h in condition 2; T_0_ = 1.5 h and T_a_ = 3.0 h in condition 3 (Table 1). The subjects were asked to report their satisfaction scores. We explored the effects of AWT on satisfaction by comparing the scores among the three conditions.

**Table 1:** Six different conditions of the experiment

Conditions	Subjects	EWT ^a^		AWT	Mimic conditions ^b^
1	Control group	T_0_ = 1.5 h	–	T_a_ = 3.0 h	T_0_ < T_a_
2	Control group	T_0_ = 1.5 h	–	T_a_ = 2.0 h	T_0_ < T_a_
3	Control group	T_0_ = 1.5 h	–	T_a_ = 1.0 h	T_0_ > T_a_
4	Experimental group	T_0_ = 1.5 h	T_1_ = 2.5 h	T_a_ = 3.0 h	T_0_ < T_1_ < T_a_
5	Experimental group	T_0_ = 1.5 h	T_1_ = 2.5 h	T_a_ = 2.0 h	T_0_ < T_a_ < T_1_
6	Experimental group	T_0_ = 1.5 h	T_1_ = 2.5 h	T_a_ = 1.0 h	T_a_ < T_0_ < T_1_

*EWT* Expected waiting time, *AWT* Actual waiting time (T_a_); T_0_, initial EWT; T_1_, second EWT; −, no data

^a^EWT was defined to be prolonged 1.0 h by unfavorable information

^b^T_0_, T_1_ and T_a_ were presented to mimic a hypothetical situation

For the experimental group, the subjects were also asked to state their initial EWT (T_0_). After UI was given, they were asked to state their second EWT (T_1_). We investigated the effects of UI on EWT by comparing T_0_ and T_1_. This study further explored the effects of an extended EWT on patient satisfaction. T_0_ was as the same as that for the control group, and T_1_ was prolonged to 2.5 h by UI. Three different hypothetical conditions were presented: T_0_ = 1.5 h, T_1_ = 2.5 h and T_a_ = 1.0 h in condition 4; T_0_ = 1.5 h, T_1_ = 2.5 h and T_a_ = 2.0 h in condition 5; T_0_ = 1.5 h, T_1_ = 2.5 h and T_a_ = 3.0 h in condition 6 (Table 1). Then, we compared the satisfaction scores between the control and experimental groups in conditions with the same T_0_ and T_a_.

### Subjects and ethics statement

For researches on prospect theory, such as in stock or house trade experiments, university students are frequently used as ideal study subjects [28–30]. This research explored the effects of EWT on patient satisfaction. In fact, apart from expectation [31], many other factors also had significant effects on patient satisfaction, including self-judged physical condition [32], lack of trust and communication between patients and doctors [33, 34], and so on. The results might be affected by these factors if the patients in the hospital were severed as study subjects. Another important problem was that the EWT could frequently change during the doctor visit process. This might hinder the implementation of this study. Hence, this study selected college students, rather than the patients in the hospital, as study subjects. Their good education background also helped them better understand our study design, which ensured a right answer to each question.

This study was performed in October 2016 using an online questionnaire survey. It enrolled 6 grade one classes from Shanghai University of Engineering Science: 3 classes were randomly assigned to the control group (*n* = 125) and 3 classes were randomly assigned to the experimental group (*n* = 132). Class teachers sent the students questionnaires online, and explained the purposes of this study and the efforts to protect privacy. Then, the students were asked to finish a questionnaire survey if they voluntarily participated in this research. A total of 257 students volunteered to participate in this research.

The design and implementation of the study and the data collection methods complied with the national guidelines [35], according to which the needs for ethics approval and informed consent were unnecessary. The study was performed in accordance with the ethical principles of the Declaration of Helsinki. Participates were informed about the purpose and privacy protection of this research at the top of the questionnaires and were notified that they could quit at any time. The data were analyzed anonymously.

### Variables

#### Demographic variables

The studied demographic variables included gender, age, and hospital visit history. The students were asked to report their hospital visit histories, because hospital visit history could help the subjects better understand the hypothetical scenario presented in the questionnaire survey. This procedure strengthened the credibility of the findings.

#### Satisfaction scores

The score was designed to measure satisfaction in response to the length of waiting time. The satisfaction scores ranged from 0 to 100: 0–25, very dissatisfied; 26–50, dissatisfied; 51–75, satisfied; 76–100, very satisfied. Satisfaction data were collected from the subjects after several simulation conditions were presented (Table 1).

### Questionnaires and tools

Our questionnaire survey included a series of hypothetical situational questions. The questionnaires consisted of two parts: the first part comprised questions that mimicked different hypothetical situations; the second part comprised individual surveys. There were 6 distinct questionnaires designed for the 6 different conditions (Table 1). They are freely available at Questionnaire Star, and the English version is available as supplementary information (Additional file 1).

First, the subjects in both the control and experimental groups were asked to report their initial EWT (T_0_). The subjects in the experimental group were further asked to given their second EWT (T_1_) after they received the UI. The difference between T_0_ and T_1_ reflected the effects of UI on EWT. Then, satisfaction scores were collected from both groups in conditions with a fixed initial EWT (T_0_ = 1.5 h) but a varying AWT (T_a_ was gradually extended from 1.0 h to 3.0 h) (Table 1). In contrast to the control group, a fixed second EWT (T_1_ = 2.5 h) was also given in the experimental group. Here, we defined that UI prolonged the EWT by 1.0 h. The differences in scores between the control and experimental groups in conditions with the same T_0_ and T_a_ reflected the effects of an extended EWT on patient satisfaction.

The questionnaire surveys were conducted in the classroom. Each class was asked to complete only one of the six different questionnaires. The class teachers sent the link for each questionnaire to the students by using WeChat. The students finished the questionnaires by clicking links on mobile phones or personal computers. The process took approximately 5–10 min.

### Data collection

The data were downloaded from Questionnaire Star. A total of 257 questionnaires were collected, 24 of which were discarded for the following reasons: (1) satisfaction scores < 0 or > 100; (2) EWT > 9.0 h (the entire workday, from 8:00 AM to 5:00 PM). The remaining 233 (90.7%) valid questionnaires were retained for further analysis.

## Statistical analysis

Statistical analysis was performed using SPSS Inc. version 17.0 software. Categorical data are described as frequencies and percentages. Quantitative data were tested for normality by the Kolmogorov-Smirnov test and the Shapiro-Wilk test. Normally distributed data and data that are basically in line with normal distribution are described as the mean ± standard deviation. Non-normally distributed data are expressed as medians (P25, P75). Parametric tests were used to test the differences among means, including the *t*-test for two groups and the one-way ANOVA test for three groups. The comparison between two medians was performed by the non-parametric Mann–Whitney test. The chi-square test was used to test the difference among ratios. *P* < 0.05 was considered significant.

## Results

### Baseline characteristics

There were 233 valid questionnaires from 233 students, including 119 in the control group and 114 in the experimental group. Their ages ranged from 17 to 22 years. Of them, 164 (70.4%) were female. Between the control and experimental groups, the average age was similar, and the gender composition ratios were not significantly different (all *P* > 0.05, Table 2). The majority (63.9%, 149/233) of the individuals, including 81 individuals in the control group and 68 individuals in the experimental group, had a hospital visit history.

**Table 2:** Baseline characteristics of the study subjects

	Control group (*n* = 119)	Experimental group (*n* = 114)
Gender		
Male	37 (31.1%)	32 (28.1%)
Female	82 (68.9%)	82 (71.9%)
Age (M [Q25, Q75]) (years)	18 [18, 19] ^a^	18 [18, 19] ^a^
Hospital visit history		
Yes	81 (68.1%)	68 (59.6%)
No	38 (31.9%)	46 (40.4%)
Expected waiting time (EWT)		
Before UI was given	120.0 [60.0, 150.0] ^a^	121.0 [90.0, 210.0] ^a^
After UI was given	–	180.0 [120.0, 240.0] ^a^

*UI* Unfavorable information; -, no data

^a^Data normality was tested by the Kolmogorov-Smirnov test and the Shapiro-Wilk test, *P* < 0.05

### Unfavorable information extends expected waiting time

Before UI was given, the median of EWT was 121.0 min in the experimental group, which was similar to that of the control group (*Z* = -1.924, *P* = 0.054) (Table 2). After UI was given, 84.2% (96/114) of individuals in the experimental group extended their EWT (Table 3), and the median of EWT was extended to 180.0 min (*Z* = -6.367, *P* < 0.001).

**Table 3:** Extension of expected waiting time (EWT) in the experimental group

	N	≤0 min	1–60 min	61–120 min	≥ 121 min
Total individuals	114	18 (15.8%)	45 (39.5%)	37 (32.5%)	14 (12.3%)
Gender					
Male	32	5 (15.6%)	18 (56.3%)	6 (18.8%)	3 (9.4%)
Female	82	13 (15.9%)	27 (32.9%) ^*^	31 (37.8%)	11 (13.4%)
Hospital visit history					
Yes	68	10 (14.7%)	25 (36.8%)	25 (36.8%)	8 (11.8%)
No	46	8 (17.4%)	20 (43.5%)	12 (26.1%)	6 (13.0%)

^*^ Ratios were compared between males and females, *P* < 0.05

After UI was given, a significant difference was found when the second EWT (T_1_) was compared between males and females in the experimental group (197.5 [121.5, 260] vs. 180 [120, 240], *Z* = -2.239, *P* = 0.025). Meanwhile, 56.3% (18/32) of the males extended their EWT by 0~60 min, and the ratio was higher than that of the females (56.3% vs. 32.9%, *χ*^*2*^ = 5.241, *P* = 0.022). Many (37.8%) females extended their EWT by 61~120 min. However, the second EWT was similar between individuals with and without a hospital visit history (187.5 [120, 240] vs. 180[120, 180], *Z* = -0.482, *P* = 0.630).

### Long actual waiting time leads to low satisfaction

To confirm the effects of AWT on patient satisfaction, the scores were compared among the 3 different conditions within the control or experimental groups respectively (Table 4). In the control group, EWT was defined as 1.5 h (T_0_ = 1.5 h). The mean of satisfaction scores was 76.0 when the AWT (T_a_) was 1.0 h, while it decreased to 51.0 when T_a_ = 2.0 h, and to 35.0 when T_a_ = 3.0 h (76.5 ± 19.9 vs. 50.1 ± 22.7 vs.38.0 ± 22.7, *F* = 32.515, *P* < 0.001) (Table 4).

**Table 4:** Satisfaction scores in the control and experimental groups

Conditions ^a^	Control group		Experimental group	
	Case	Score	Case	Score
T_0_ = 1.5 h (T_1_ = 2.5 h) and T_a_ = 1.0 h	41	76.5 ± 19.9 ^b^	37	80.9 ± 15.6 ^b^
T_0_ = 1.5 h (T_1_ = 2.5 h) and T_a_ = 2.0 h	40	50.1 ± 22.7	44	66.7 ± 19.9 ^c^
T_0_ = 1.5 h (T_1_ = 2.5 h) and T_a_ = 3.0 h	38	38.0 ± 22.7	33	41.9 ± 22.7

T_0_, expected waiting time (EWT) before unfavorable information (UI) was given; T_1_, EWT after UI was given; T_a_, actual waiting time

^a^EWT was prolonged 1.0 h by UI in the experimental group (T_1_ = 2.5 h)

^b^Data normality was tested by the Kolmogorov-Smirnov test, *P* > 0.05, by the Shapiro-Wilk test, *P* < 0.05

^c^Scores in the control group vs. scores in the experimental group, *P* < 0.05

In the experimental group, we defined that the EWT was prolonged to 2.5 h by UI (T_1_ = 2.5 h). The mean of satisfaction scores was 80.9 when T_a_ = 1.0 h, while it decreased to 70.0 when T_a_ = 2.0 h, and to 42.0 when T_a_ = 3.0 h (80.9 ± 15.6 vs. 66.7 ± 19.9 vs. 41.9 ± 22.7, *F* = 35.462, *P* < 0.001).

### Extending the expected waiting time improves satisfaction

To analyze the effects of EWT on patient satisfaction, the scores were compared between the control and experimental groups under conditions with the same AWT (T_a_) and initial EWT (T_0_) (Table 4). A significant difference was only found when T_a_ = 2.0 h and T_0_ = 1.5 h (*t* = − 3.568, *P* = 0.001), but not when T_a_ = 3.0 h and T_0_ = 1.5 h (*t* = − 0.718, *P* = 0.475) or when T_a_ = 1.0 h and T_0_ = 1.5 h (*t* = − 1.088, *P* = 0.280).

When T_a_ = 2.0 h and T_0_ = 1.5 h, 77.3% (34/44) of individuals felt “satisfied” or “very satisfied” after a longer EWT (T_1_) was given in the experimental group (Table 5). The ratio was higher than that of the control group (34/44 vs. 21/40, *χ*^*2*^ = 5.688, *P* = 0.017). For the remaining two conditions, the ratios of individuals feeling “satisfied” or “very satisfied” were similar between the control and experimental groups (all *P* > 0.05). However, fewer individuals felt “very dissatisfied” in experimental group than in the control group when T_a_ = 3.0 h and T_0_ = 1.5 h (21.2% vs. 44.7%, *χ*^*2*^ = 4.368, *P* = 0.037).

**Table 5:** The interval distribution of satisfaction scores in the control and experimental groups

	N	Very dissatisfied	Dissatisfied	Satisfied	Very satisfied
T_0_ = 1.5 h and Ta =1.0 h					
Control group	41	1 (2.4%)	2 (4.9%)	17 (41.5%)	21 (51.2%)
Experimental group (T_1_ = 2.5 h)	37	1 (2.7%)	0 (0.0%)	12 (29.7%)	25 (67.6%)
T_0_ = 1.5 h and T_a_ = 2.0 h					
Control group	40	6 (15.0%)	13 (32.5%)	16 (40.0%)	5 (12.5%)
Experimental group (T_1_ = 2.5 h)	44	1 (2.3%) ^a^	9 (20.5%)	19 (43.2%)	15 (34.1%) ^a^
T_0_ = 1.5 h and T_a_ = 3.0 h					
Control group	38	17 (44.7%)	10 (26.3%)	9 (23.7%)	2 (5.3%)
Experimental group (T_1_ = 2.5 h)	33	7 (21.2%) ^a^	17 (51.5%) ^a^	7 (21.2%)	2 (6.1%)

^a^Satisfaction scores in control group vs. satisfaction scores in the experimental group, *P* < 0.05

## Discussion

Due to the shortage of medical resources in China, patients in outpatient departments are kept waiting for long times to see their doctors, especially in the large hospitals. Long AWT results in low satisfaction [36–38]. To improve satisfaction, hospitals and researchers have implemented several methods for reducing the AWT [7–16]. However, patients still have to wait a long time in outpatient departments of large hospitals in China. This study investigated the impacts of UI on EWT, and further explored the effects of an extended EWT on satisfaction. We verified that a long AWT could result in low satisfaction, especially when the EWT was fixed. We also found that the EWT could be extended by UI, and that an extended EWT could improve satisfaction. This research shed a new light on how to improve patient satisfaction by extending the EWT instead of shortening the AWT.

Consistent with previous research [37], this study confirmed that a longer AWT resulted in lower satisfaction in both the control and experimental groups. Both groups had a fixed EWT. However, shortening the AWT did not always improve satisfaction according to our data. We found that the satisfaction scores were similar between the control group when T_a_ = 2.0 h and the experimental group when T_a_ = 3.0 h (*P* = 0.087). This could be attributed to the change in the EWT: EWT =1.5 h in the control group, but 2.5 h in the experimental group. Therefore, we inferred that shortening the AWT could improve patient satisfaction, especially when the EWT was fixed. This highlighted the important effects of EWT on individuals’ satisfaction.

EWT was found to be greatly extended after UI was given. It indicated that the individuals in the experimental group planned to spend more waiting time in the outpatient departments. In prospect theory, whether an individual feeling gain or loss depends on the reference point [39, 40]. EWT could severe a reference point. The individuals might feel better by adjusting their reference point according to UI [41–43]. Therefore, UI could be used to extend EWT in outpatient departments. We also found that gender had great effects on EWT. Given the same UI, females extended their EWT to a greater extent than males did. This could be attributed to the fact that females were more risk averse than males [44]. Females extended their EWT longer than males to avoid the uncomfortable feeling caused by a long AWT. Hospital visit history did not have significant effects on EWT: the responses to UI were similar between subjects with and without a hospital visit history.

In this study, we found that patient satisfaction could be improved by an extended EWT. The improvement of satisfaction could be attributed to the reference point’s determination of the individual’s value judgment and risk behavior [21]. Differences in reference point led to different judgments [45]. For the same result, some individuals will fell satisfied, but others will fell dissatisfied, because they had different reference points [46]. When T_a_ = 2.0 h, the individuals in the control group felt loss because the AWT was longer than the EWT, while the subjects in the experimental group experienced a gain because the AWT was shorter than the extended EWT. This improvement in satisfaction could be attributed to that the subjects’ change in utility from the original loss area (EWT < AWT) to the gain area (EWT > AWT). However, the extended EWT did not cause a change in the subjects’ utility when T_a_ = 1.0 h and when T_a_ = 3.0 h. When T_a_ = 1.0 h, the individuals in both groups were in the gain area. Extending EWT only resulted in a slight increase in proportion of individuals who reported feeling “very satisfied” (*P* = 0.143). When T_a_ = 3.0 h, the individuals in both groups were in the loss area. Extending EWT led to a great decrease in the proportion of individuals who felt “very dissatisfied”. According to the perspective of loss aversion in prospect theory, a change of one unit in the loss area has greater impact on value than a similar change in the gain area [21]. Hence, we concluded that extending EWT could improve satisfaction.

This study provides an alternative for improving patient satisfaction aside from reducing the AWT. It will work at many healthcare contexts, especially in emergency situations, when long AWT is inevitable or shorten AWT becomes impossible. The medical administrators can establish an early warning mechanism, which can inform the outpatients of a large number of waiting patients in real-time by electronic screen, loudspeaker, or other approaches at the peak of patient visits. The subjects will extend their EWT according to the number of waiting patients. It can be used to improve patient satisfaction and helps to avoid negative emotions arising from a long AWT.

### Limitations and strengths

The major limitation of this study was that the study subjects were university students, but not patients in the hospital. It might cause response bias, because the subjects finished the questionnaire in the school rather than a clinic. The process was different from doctor visit. However, each subject was a potential patient. The response reflected their real expectations for doctor visit, although it would affect by many factors during the doctor visit process. Furthermore, the majority of enrolled university students had a hospital visit history, which increased the credibility of our findings. The aims of the study were to investigate the effects of UI on EWT and to further explore the effects of an extended EWT on patient satisfaction. Our design could avoid the effects of other factors by selecting university students as the study subjects. Second, the absence of monetary incentives for participations might result in unrealistic answers. However, it has been proven that there are no differences in decision making between participants who do and do not receive monetary payments [47]. This study found that UI could extend EWT, and that an extended EWT could improve patient satisfaction. It represents a new step toward improving patient satisfaction by adjusting the EWT but not the AWT.

## Conclusions

Using a questionnaire survey, this study confirmed that a long AWT resulted in low satisfaction, especially when the EWT was fixed. Second, it showed that EWT could be greatly extended by UI, and that gender, but not hospital visit history, had important effects on the extension of EWT. Third, we found that an extended EWT could improve satisfaction; however, significant improvement in satisfaction score was only found when the individual’s utility changed from the original loss area to the gain area by extending the EWT. This study provided a new idea for improving satisfaction among outpatients in the large hospitals in China by extending the EWT instead of shortening the AWT. Future researches could investigate the patient satisfaction before and after the release of UI during peak hours.

## Additional file

Additional file 1: Six distinct questionnaires in English. (DOC 58 kb)

## Data Availability

The datasets used and/or analyzed during the current study are available from the corresponding author on reasonable request.

## Abbreviations

AWT: Actual waiting time
EWT: Expected waiting time
UI: Unfavorable information
