# Supplementary material for: Improving outpatient satisfaction by extending expected waiting time
Source: BMC Health Serv Res. 2019 Aug 13;19:565. doi: 10.1186/s12913-019-4408-3 (PMC6693195; doi:10.1186/s12913-019-4408-3)
Supplement: Supplementary file 1 — Six distinct questionnaires in English. (DOC 58 kb) [file 12913_2019_4408_MOESM1_ESM.doc]

**Six distinct questionnaires in English.**

**Condition 1**

Welcome to this experiment! Please make your choice according to your first feeling. This experiment is only for academic research. There is no right or wrong answers. Please read the following questions carefully:

1. If you felt discomfort today and were preparing to see a doctor at a large hospital nearby, how long do you expect to wait from queuing to leaving the hospital gate? ___ hours ____minutes

2. If today's waiting time is longer than your expected waiting time, please give a satisfaction score of your waiting time.______

(0-25, very dissatisfied; 26-50, dissatisfied; 51-75, satisfied; 76-100, very satisfied)

3. If you expect to wait for 1.5 hours. And you actually wait for 3 hours today (1.5 hours longer than your second excepted waiting time). Please give a satisfaction score of your waiting time.______

(0-25, very dissatisfied; 26-50, dissatisfied; 51-75, satisfied; 76-100, very satisfied) 4. Have you had any experience of visiting a local large hospital?

□Yes □No

5. Sex

□Male □Female

6. Age

____years

**Condition 2**

Welcome to this experiment! Please make your choice according to your first feeling. This experiment is only for academic research. There is no right or wrong answers. Please read the following questions carefully:

1. If you felt discomfort today and were preparing to see a doctor at a large hospital nearby, how long do you expect to wait from queuing to leaving the hospital gate? ___ hours ____minutes

2. If today's waiting time is longer than your expected waiting time, please give a satisfaction score of your waiting time.______

(0-25, very dissatisfied; 26-50, dissatisfied; 51-75, satisfied; 76-100, very satisfied)

3. If you expect to wait for 1.5 hours. And you actually wait for 2 hours today (0.5 hours longer than your second excepted waiting time). Please give a satisfaction score of your waiting time.______

(0-25, very dissatisfied; 26-50, dissatisfied; 51-75, satisfied; 76-100, very satisfied) 4. Have you had any experience of visiting a local large hospital?

□Yes □No

5. Sex

□Male □Female

6. Age

____years

**Condition 3**

Welcome to this experiment! Please make your choice according to your first feeling. This experiment is only for academic research. There is no right or wrong answers. Please read the following questions carefully:

1. If you felt discomfort today and were preparing to see a doctor at a large hospital nearby, how long do you expect to wait from queuing to leaving the hospital gate? ___ hours ____minutes

2. If today's waiting time is shorter than your expected waiting time, please give a satisfaction score of your waiting time.______

(0-25, very dissatisfied; 26-50, dissatisfied; 51-75, satisfied; 76-100, very satisfied)

3. If you expect to wait for 1.5 hours. And you actually wait for 1 hour today (0.5 hours shorter than your second excepted waiting time). Please give a satisfaction score of your waiting time.______

(0-25, very dissatisfied; 26-50, dissatisfied; 51-75, satisfied; 76-100, very satisfied) 4. Have you had any experience of visiting a local large hospital?

□Yes □No

5. Sex

□Male □Female

6. Age

____years

**Condition 4**

Welcome to this experiment! Please make your choice according to your first feeling. This experiment is only for academic research. There is no right or wrong answers. Please read the following questions carefully:

1. If you felt discomfort today and were preparing to see a doctor at a large hospital nearby, how long do you expect to wait from queuing to leaving the hospital gate? ___ hours ____minutes

2. You are informed that there are many patients in the outpatient department today when you enter the hospital. How long do you expect to wait from queuing to leaving the hospital gate? ___ hours ____minutes

3. If today's waiting time is longer than your second expected waiting time, please give a satisfaction score of your waiting time.______

(0-25, very dissatisfied; 26-50, dissatisfied; 51-75, satisfied; 76-100, very satisfied)

4. If you expect to wait for 1.5 hours firstly, after you know that there are many patients in the outpatient department today, you expect it will take 2.5 hours. And you actually wait for 3 hours today (0.5 hours longer than your second excepted waiting time). Please give a satisfaction score of your waiting time.______

(0-25, very dissatisfied; 26-50, dissatisfied; 51-75, satisfied; 76-100, very satisfied) 5. Have you had any experience of visiting a local large hospital?

□Yes □No

6. Sex

□Male □Female

7. Age

____years

**Condition 5**

Welcome to this experiment! Please make your choice according to your first feeling. This experiment is only for academic research. There is no right or wrong answers. Please read the following questions carefully:

1. If you felt discomfort today and were preparing to see a doctor at a large hospital nearby, how long do you expect to wait from queuing to leaving the hospital gate? ___ hours ____minutes

2. You are informed that there are many patients in the outpatient department today when you enter the hospital. How long do you expect to wait from queuing to leaving the hospital gate? ___ hours ____minutes

3. If today's waiting time is longer than your first expected waiting time and shorter than your second expected waiting time, please give a satisfaction score of your waiting time.______

(0-25, very dissatisfied; 26-50, dissatisfied; 51-75, satisfied; 76-100, very satisfied)

4. If you expect to wait for 1.5 hours firstly, after you know that there are many patients in the outpatient department today, you expect it will take 2.5 hours. And you actually wait for 2 hours today (0.5 hours shorter than your second excepted waiting time). Please give a satisfaction score of your waiting time.______

(0-25, very dissatisfied; 26-50, dissatisfied; 51-75, satisfied; 76-100, very satisfied) 5. Have you had any experience of visiting a local large hospital?

□Yes □No

6. Sex

□Male □Female

7. Age

____years

**Condition 6**

Welcome to this experiment! Please make your choice according to your first feeling. This experiment is only for academic research. There is no right or wrong answers. Please read the following questions carefully:

1. If you felt discomfort today and were preparing to see a doctor at a large hospital nearby, how long do you expect to wait from queuing to leaving the hospital gate? ___ hours ____minutes

2. You are informed that there are many patients in the outpatient department today when you enter the hospital. How long do you expect to wait from queuing to leaving the hospital gate? ___ hours ____minutes

3. If today's waiting time is shorter than your first expected waiting time, please gives a satisfaction score of your waiting time.______

(0-25, very dissatisfied; 26-50, dissatisfied; 51-75, satisfied; 76-100, very satisfied)

4. If you expect to wait for 1.5 hours firstly, after you know that there are many patients in the outpatient department today, you expect it will take 2.5 hours. And you actually wait for 1 hour today (1.5 hours shorter than your second excepted waiting time). Please give a satisfaction score of your waiting time.______

(0-25, very dissatisfied; 26-50, dissatisfied; 51-75, satisfied; 76-100, very satisfied)

5. Have you had any experience of visiting a local large hospital?

□Yes □No

6. Sex

□Male □Female

7. Age

____years
